# Comparative Analysis and Classification of Cassette Exons and Constitutive Exons

**DOI:** 10.1155/2017/7323508

**Published:** 2017-12-04

**Authors:** Ying Cui, Meng Cai, H. Eugene Stanley

**Affiliations:** ^1^School of Mechano-Electronic Engineering, Xidian University, Xi'an 710071, China; ^2^Center for Polymer Studies and Department of Physics, Boston University, Boston, MA 02215, USA; ^3^School of Economics and Management, Xidian University, Xi'an 710071, China

## Abstract

Alternative splicing (AS) is a major engine that drives proteome diversity in mammalian genomes and is a widespread cause of human hereditary diseases. More than 95% of genes in the human genome are alternatively spliced, and the most common type of AS is the cassette exon. Recent discoveries have demonstrated that the cassette exon plays an important role in genetic diseases. To discover the formation mechanism of cassette exon events, we statistically analyze cassette exons and find that cassette exon events are strongly influenced by individual exons that are smaller in size and that have a lower GC content, more codon terminations, and weaker splice sites. We propose an improved random-forest-based hybrid method of distinguishing cassette exons from constitutive exons. Our method achieves a high accuracy in classifying cassette exons and constitutive exons and is verified to outperform previous approaches. It is anticipated that this study will facilitate a better understanding of the underlying mechanisms in cassette exons.

## 1. Introduction

In most eukaryotic organisms, gene expression is regulated by alternative splicing (AS), which is the major posttranscriptional mechanism that promotes biological complexity. AS is a biological process in which one gene produces a variety of transcript isoforms through the distinct selection of splice sites during pre-mRNA splicing. AS plays an important regulatory role in proteome diversity [1, 2]. Up to 95% of human genes are alternatively spliced in order to encode proteins with different functions [3], and approximately 15% of human hereditary diseases and cancers are caused by AS [4].

Cassette exon splicing, also known as exon skipping, is the most prevalent form of alternative splicing in the human genome and accounts for 50 to 60 percent of all alternatively spliced events [5]. A cassette exon is a splicing event in which an intervening exon between two other exons from the mature mRNA sequence can be either included or skipped in order to generate two distinct protein isoforms. A number of recent discoveries have concluded that the cassette exon is closely associated with a broad range of human diseases [6–11] such as renal cancer [9], neuromuscular diseases [10], and hearing loss [11]. Cassette exons have also been employed as a therapeutic strategy for producing the required proteins for genetic diseases [10, 12] such as the congenital myasthenia syndrome [12]. Despite its importance, because of its complexity, the cassette exon mechanism is not fully understood, and because of the limited availability of accurate computational tools, genome-wide detection of cassette exons remains a major challenge. Thus, we focus our attention here on a comparative analysis of the sequence features of cassette exons and constitutive exons in order to find the regulatory factors that contribute to cassette exon events. We also aim to construct a classification model that can distinguish between cassette exons and constitutive exons.

Various machine learning approaches are used in cassette exons identification [13–16]. Dror et al. used support vector machine (SVM), which is one of the classical machine learning methods, to classify cassette exons and constitutive exons conserved between human and mouse [13]. This research is highly dependent on conservation-based features, and thus its application on cassette exons detection is constrained to exons conserved between human and mouse genomes. Su et al. also proposed an SVM-based approach, which uses two classifiers [14]. The first classifier distinguishes authentic exons from pseudo exons, and the second classifier distinguishes cryptic exons from constitutive and skipped exons. This method did not use conservation information and therefore can be used more widely and easily. However, it only can achieve about 70% accuracy. Li et al. introduced an AdaBoost-based method to identify cassette exons and achieved 77.83% accuracy [15]. Recently, a method combining a gene expression programming (GEP) algorithm and a random forest (RF) model was proposed for identifying cassette exons in the human genome [16] and achieved a significantly higher accuracy (90.87%) than previous studies. Although this hybrid program is a contribution that allows the investigation of cassette exons in human genes, its results still need improving. This GEP+RF method [16] also cannot speculate on the regulators of the formation mechanism of cassette exons events.

To understand why cassette exons occur and what kind of exon is more likely to be skipped, we here analyze and compare the sequence features of cassette exons with the constitutive exons in the human genome. We find that exons with shorter length, weaker splice sites, lower GC content, and more termination codons are more likely to be skipped during the splicing process. We propose a hybrid classification method based on an improved random forest model for distinguishing cassette exons in the human genome from constitutive exons. Computational simulation results indicate that our approach more accurately identifies cassette exons than previous methods.

## 2. Materials and Methods

### 2.1. Datasets

We use the HEXEvent database, which provides a list of human internal exons and reports all known splice events, as our source of alternative splicing information [17]. We downloaded the complete list of cassette exons and constitutive exons of the first human chromosome from the HEXEvent database. We also downloaded sequences of the first human chromosome from the UCSC Genome Browser [18] and extracted cassette exon and constitutive exon sequences using the lists we obtained from HEXEvent. Only exons without any other kind of AS events supported by the ESTs were used for analysis, and this produced a final dataset of 3120 cassette exons and 7316 constitutively spliced exons.

### 2.2. Feature Extraction and Analysis

Here, we compute and comparatively analyze the sequence features of cassette exons and constitutive exons to find out how they differ and to extract classification features for use in the next step. We compute 91 features including length, nucleotide composition (mononucleotide, dinucleotide, and trinucleotide), GC content, termination codon frequency, and splice site strength, and we use the *t*-test to analyze the differences between the two groups of data.

Although splice site strength signal is typically modeled using methods such as the first-order Markov model, the weight matrix model, and the maximum entropy (MaxEnt) model [19], the first-order Markov model disregards connections between nonadjacent positions and the weight matrix model is hindered by the hypothesis that different positions are independent. We thus use MaxEnt model to determine the strength of the splice site signal.

The MaxEnt model obtains the most likely distribution function of observables from statistical systems by maximizing the entropy under a set of constraints that model the incomplete information about the target distribution. The MaxEnt model consists of two distributions: the scilicet signal model (*P*^+^(*x*)) and the decoy probability distribution  (*P*^−^(*x*)).

Let *p* denote the unknown probability distribution. For a specific DNA sequence* x*, *p*(*x*) represents its probability. Let $\hat{p}$ be the approximation of *p*, where the entropy of $\hat{p}$ is defined as

(1) $$\begin{array}{c} H\left(\;\hat{p}\;\right)=-\sum \hat{p}\left(\;x\;\right)\log_{2}\left(\;\hat{p}\left(\;x\;\right)\;\right). \end{array}$$

For a given sequence* x*, the signal strength of *x* is

(2) $$\begin{array}{c} L\left(\;X=x\;\right)=\frac{p^{+}\left(X=x\right)}{p^{-}\left(X=x\right)}. \end{array}$$

Here, *p*^+^(*X* = *x*) and *p*^−^(*X* = *x*) are the probability of *x* from the distribution of signals (+) and decoys (−), respectively.

**Figure 1:**
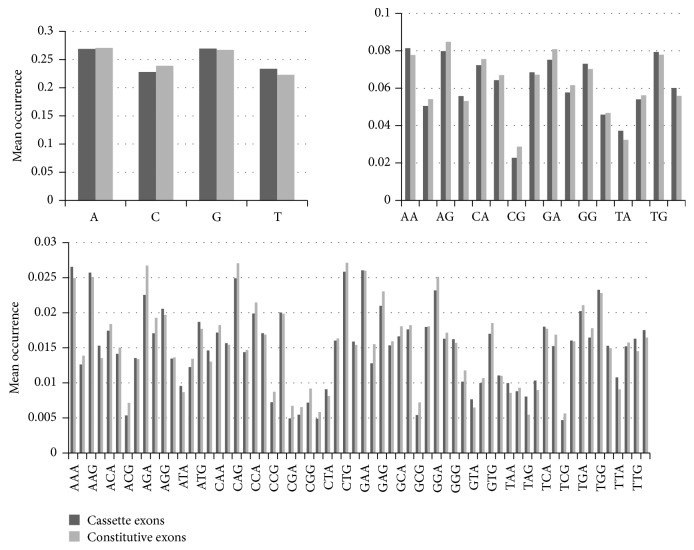
Mean occurrence of mononucleotide, dinucleotide, and trinucleotide.

Table 1 and Figure 1 show the feature analysis results. We find that cassette exons are usually shorter in size and have a lower GC content, more termination codons, and weaker splice sites than constitutive exons. The strength of a splice site determines the skipping level in AS, so weak splice sites are suboptimal for the splicing mechanism. Apparently, short length, abundance of terminal codons, and weak splice signal hinder the ability of the splicing mechanism to recognize these exons, and the result is exon skipping.

### 2.3. Classifiers

We employ five binary classifiers to distinguish cassette exons from constitutive exons based on the 91 extracted features.

#### 2.3.1. KNN Classifier

The *k*-nearest neighbors (KNN) algorithm is a linear pattern recognition method used for classification and regression [20]. For classification, KNN compares a test tuple and a collection of labeled examples in a training set. Each new sample in the prediction set is classified according to the class of the majority of its *k*-nearest neighbors in the training set. Parameter “*K*” is the number of neighbors used to determine the class, and it strongly influences the identification rate of the KNN model.

#### 2.3.2. SVM Classifier

The support vector machine (SVM) is a supervised machine learning algorithm based on statistical learning theory [21]. SVM is primarily used to solve classification and regression problems and has been successfully applied to bioinformatics tasks such as alternative splice sites identification and diagnostic method of diabetes [22, 23]. SVM uses a nonlinear mapping function to map original data into a high-dimensional feature space. It then constructs a hyperplane to be the surface that discriminates between positive and negative data.

#### 2.3.3. RF Classifier

The random forest (RF) algorithm is an ensemble machine learning method developed by Breiman [24]. It has been widely applied to prediction problems in bioinformatics [25–27]. The RF algorithm consists of multibase tree-structured classifiers such as CART (classification and regression tree), and it is robust to noise, is not hindered by overfitting, and is computationally feasible. By applying CART as a base classifier, RF collects the outputs of all decision trees, tallies the result, and uses the result to classify the sample.

#### 2.3.4. CF Classifier

The majority-voting rule in the traditional RF algorithm can cause minority categories to be misclassified. Thus, an improved RF function, the CForest (CF) function, has been proposed [28]. Unlike the standard RF algorithm based on the CART with its unfair splitting criterion, the CForest function uses a conditional inference framework to create an unbiased tree base classification model.

In the CForest algorithm, conditional inference trees are used to evaluate the association between the predictor variable and the response. The CForest algorithm works as follows. (i) The algorithm tests the global null hypothesis of independence between any of the input variables and the response and continues until this hypothesis is accepted. If it is not, the input variable with the strongest connection to the response is selected. (ii) The selected predictor variable is split into two disjoint sets. (iii) Steps (i) and (ii) are repeated.

The most important part of a forest framework is the splitting objective function. In the traditional RF, the most common measures are the information gain and the Gini impurity, which are biased towards relevant predictor variables. In response, Strobl et al. [29] proposed adding a conditional permutation importance scheme to the CForest framework. This permutation importance scheme uses a fitted forest model to partition the entire feature space, and it can be used to demonstrate the influence of a variable and to compute the importance of its permutation, conditional on all types of correlated covariates.

The CForest framework provides unbiased variable importance measurements and uses conditional permutation for feature selection. Here, the importance of a predictor variable is measured by comparing the accuracy of the prediction before and after the permutation.

Let ${\overset{-}{B}}^{\left(t\right)}$ represent the out-of-bag (oob) sample for a tree* t*, with *t* ∈ {1,…, *ntree*}. Here,* ntree* is the number of trees in the forest. The oob-prediction accuracy in tree *t* before the permutation is

(3) $$\begin{array}{c} \frac{\sum_{i}^{}{\overset{-}{B}}^{\left(t\right)}I\left(y_{i}={\hat{y}}_{i}^{\left(t\right)}\right)}{\left|{\overset{-}{B}}^{\left(t\right)}\right|}, \end{array}$$

where ${\hat{y}}_{i}^{\left(t\right)}=f^{\left(t\right)}\left(x_{i}\right)$ is the predicted class for observation *i* before permutation.

After permuting the value of *X*_*j*_, the new accuracy is

(4) $$\begin{array}{c} \frac{\sum_{i}^{}{\overset{-}{B}}^{\left(t\right)}I\left(y_{i}={\hat{y}}_{i,\left.\pi_{j}\right|Z}^{\left(t\right)}\right)}{\left|{\overset{-}{B}}^{\left(t\right)}\right|}, \end{array}$$

where *Z* is the remaining predictor variables *Z* = *X*_1_,…, *X*_*j*−1_, *X*_*j*+1_,….

Then, the variable importance of *X*_*j*_ in tree *t* can be expressed:

(5) VItXj=∑iB−tIyi=y^itB−t−∑iB−tIyi=y^i,πjZtB−t.

Finally, the importance of each variable *X*_*j*_ for the forest is calculated as an average over all trees:

(6) $$\begin{array}{c} VI\left(\;X_{j}\;\right)=\frac{\sum_{t=1}^{ntree}{VI}^{\left(t\right)}\left(X_{j}\right)}{ntree}. \end{array}$$

#### 2.3.5. XGBoost Classifier

EXtreme Gradient Boosting (XGBoost) [30] is a scalable machine learning system for tree boosting and is one of the most popular machine learning methods in recent years. Based on the principle of gradient boosting machines proposed by Friedman [31], XGBoost produces a boosting ensemble of weak classification trees by optimizing a differentiable loss function with gradient descent algorithm. XGBoost deals with overfitting and is computationally efficient.

### 2.4. Performance Assessment

The *k*-fold cross-validation technique is commonly used to measure the performance of a classifier as it overcomes the problem of overfitting. The *k*-fold cross-validation method does not use the entire dataset to train the model but randomly splits the data into *k* equal-sized smaller subsets. Of the *k* subsets, a single subset is retained to be the validation data for testing the model, and the remaining *k* − 1 subsets are used as training data. The cross-validation process is then repeated *k* times (the folds), with each of the *k* subsets used once as test data to assess the performance of the model. We can then use the average of *k* results from the fold to assess the performance of the constructed model.

A receiver operating characteristic (ROC) curve is a plot of the performance of a binary classifier system that shows the true positive rate against the false positive rate for different cut points. We often use this plot to calculate the area under a curve (AUC) to measure the performance of a classification model. The larger the area under the curve (a perfect prediction will make AUC equal to 1), the better the predictive accuracy of the model.

The evaluation indicators we use to measure performance are sensitivity, specificity, and total accuracy:

(7) Sn=TPTP+FN,Sp=TNTN+FP,TA=TP+TNTP+TN+FN+FP,

where TP is the number of correctly recognized positives, FN is the number of positives recognized as negatives, FP signifies the number of negatives recognized as positives, and TN is the number of correctly recognized negatives. Positives are cassette exons and negatives are constitutive exons.

## 3. Results

### 3.1. Sample Preparation

The 91 extracted sequence features (listed in Table 2) are combined in tandem to create an input classifier vector. To eliminate the negative effects of magnitude difference in value between different features, we scale the feature values to a range of −1 and 1. Cassette exons and constitutive exons are considered to be positive and negative samples, respectively. In the 10-fold cross-validation, the whole dataset is randomly and equally divided into ten sets. Each set is used as a testing set, and the remaining nine are used for training. We use a total of ten testing sets, and each training set is nine times the size of its corresponding testing set.

### 3.2. Performance of Different Classifiers

To highlight the good performance in discrimination of cassette exons and constitutive exons according to sequence features, we attempted to compare the classification results from KNN, SVM, RF, CF, and XGBoost approaches in this work. In the conduction of classifier model, parameter tuning is essential for building a binary-class model with high predictive accuracy and stability. In each classifier, parameters were optimized according to the prediction performance they eventually achieve. A parameter will be settled when it reaches the best prediction performance.

In the KNN model, we search the optimal parameter *K* value by using a 10-fold cross-validation. We estimate the predictive errors for a given set of *K* values using cross-validation and select the *K* value that allows the subsequent KNN classification to yield optimal results. Thus, we optimize 20* K* values (*K* = 1, 2, … , 20) using the 10-fold cross-validation. The KNN classifier achieves optimal performance when *K* = 7.

In the SVM model, we examine the radial basis function (RBF) kernel, the linear kernel, the polynomial kernel, and the sigmoid kernel. Each kernel function has several parameters, and properly tuning them significantly affects the classification performance of the SVM model. We use a grid-search technique and 10-fold cross-validation to search the optimal parameter values of SVM using the four kernels.  Table 3 supplies the predictive performance results of the four kernels in the SVM model. Of the four, the RBF kernel shows the best predictive accuracy, and we choose it to be the basic kernel function of the SVM classifier. There are two important parameters associated with RBF kernels: *C* and *g*. We use a grid search and 10-fold cross-validation and find the optimal (*C*, *g*) pair to be (25, 0.000012).

In the random forest model, two parameters play an important role—*ntree* and* mtry*. The* ntree* parameter is the number of trees in the forest, and the* mtry* parameter is the number of variables available for splitting at each tree node. The forest error rate depends on two things: (i) the correlation between any two trees in the forest (increasing the correlation increases the error rate) and (ii) the strength of each individual tree in the forest (a tree with a low error rate is a strong classifier, and increasing the strength of the individual trees decreases the forest error rate). A larger number of trees produce a more stable model and covariate importance estimates but require more memory and a longer run time. Reducing* mtry* reduces both the correlation and the strength. Increasing it increases both. Somewhere in between is an “optimal”* mtry* range. We use a grid search and 10-fold cross-validation to find the optimal* ntree* and* mtry*. We find that the RF classifier performs best when* ntree * = 1000 and* mtry * = 91.

CForest parameter optimization is similar to the random forest. A grid search with 10-fold cross-validation is used to determine the best* ntree* and* mtry*. The optimal* (ntree*,* mtry)* pair is (1050, 10).

In the XGBoost model, the type of model is set as tree-based models. We mainly tuned two parameters,* eta* and* max_depth*. The* eta* is analogous to learning rate, which is the step size shrinkage used when getting the weights of new features after each boosting step to prevent overfitting. The max_depth is the maximum depth of a tree, which is used to control overfitting, as higher depth will make the model more complex. We use a grid search and 10-fold cross-validation to tune these two parameters and the optimal values are* eta* = 0.5 and* max_depth* = 4.  Table 4 displays all the parameter optimization details of the KNN, SVM, RF, CF, and XGBoost models.

**Figure 2:**
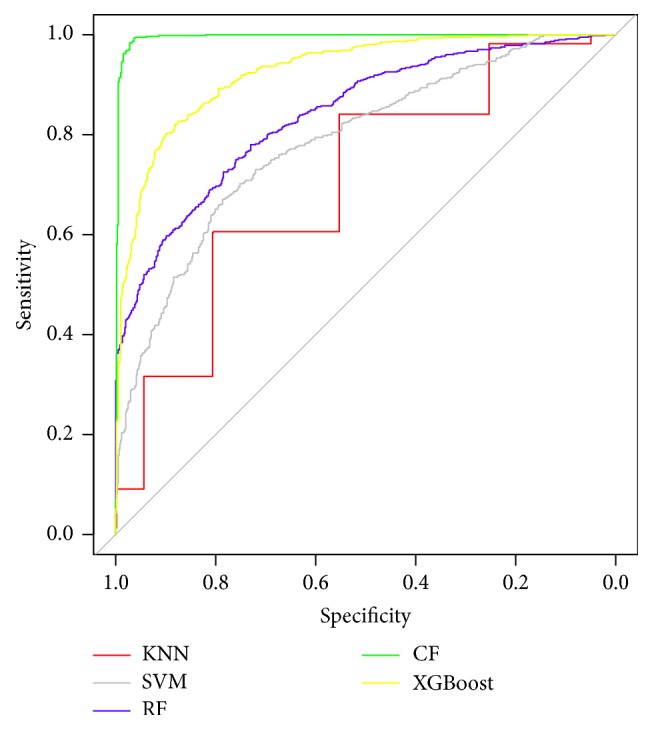
ROC curves of different classifiers.

Using the optimal parameters, we construct five classifier models for identifying cassette exons based on KNN, SVM, RF, CF, and XGBoost models. We use sensitivity, specificity, total accuracy, and AUC to compare their predictive performances (shown in Table 5), and Figure 2 shows the ROC curves of different classifiers represented by different colors. It can be observed that CForest classifier significantly outperforms other classifiers. As another tree-based model, XGBoost wins the second place. We can see from Table 5 that there is an obvious imbalance between sensitivity and specificity in the RF classifier (Sn = 71.49%, Sp = 78.72%), where Sn is notably lower than Sp, indicating that RF classifier can distinguish true constitutive exons from cassette exons but is not so effective in recognizing true cassette exons. This phenomenon can also be seen in KNN classifier (Sn = 67.03%, Sp = 75.57%), another majority-voting principle based model. In contrast, CF classifier can do both; Sp is only 1.72 percent higher than Sn. This is the case because CForest classifier provides an unbiased measure of variable importance.

Generally, the nonlinear models (SVM, RF, CF, and XGBoost) are superior to the linear model (KNN), and the CForest model is the best. Theoretically, the nonlinear method performs better than the linear method when applied to self-learning and self-adjusting. The nonlinear model has a simpler structure and a higher classification performance. In a forest-based model, the splitting rule plays an essential role in the prediction accuracy. Traditional RF uses the majority-voting rule to split, which shows a bias towards relevant predictor variables. This unfair splitting criterion tends to make the minority categories more likely to be misclassified. In response to this limitation, CForest is established by unbiased base classification trees based on a conditional inference framework. The CForest model assembles unbiased base classification trees into a conditional inference framework and thus eliminates the errors caused by the majority-voting rule used in the KNN, RF, and XGBoost models. In our case, there is a notable imbalance of the amount between cassette exons and constitutive exons. Thus, the CForest model is theoretically superior and exhibits a better predictive performance than the other classifiers.

### 3.3. Feature Importance

**Figure 3:**
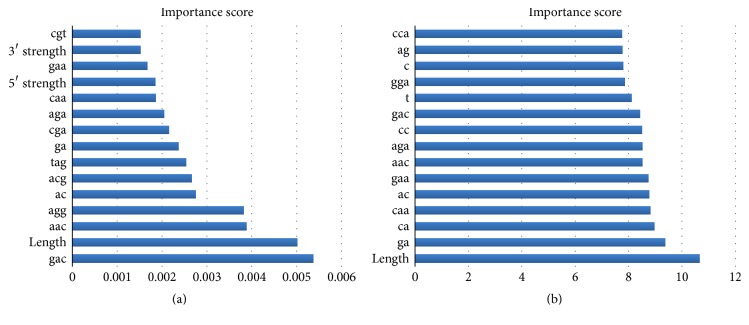
Importance rank of features (top 15) in different classification models. (a) CForest, (b) random forest.

The CForest model provides an unbiased measure of variable importance, which can used to evaluate the importance of the features applied in the classification. We calculate the importance scores of all sequence features and rank the features according to their importance scores to explore the most essential features that predict cassette exons in our model.  Figure 3(a) shows the top 15 features ranked according to their importance scores generated by the CForest classifier. The rank of a feature indicates its importance when discriminating cassette exons from constitutive exons. We can observe from Figure 3(a) that, in the top 15 features, 12 of them are dinucleotides and trinucleotides. Moreover, 8 of the 12 motifs (“gac,” “agg,” “acg,” “tag,” “ga,” “cga,” “aga,” and “gaa”) contain “ag” or “ga.” This indicates that “ag” and “ga” play a role in the occurrence of cassette exon events. Additionally, it is speculated that length, 5′ splice site strength, and 3′ splice site strength are the regulators of cassette exon events in the human genome. In Figure 3(b), we display the top 15 features ranked according to their importance scores generated in the random forest classification model. Random forest provides two measures to evaluate feature importance; here, we use the most common one known as Gini impurity. It can be seen from Figure 3(b) that, in the RF classifier, the most important feature is the length, which is the second important feature in CF classifier. Similar to CF, 6 of the top 15 features motifs (“ag,” “gga,” “gac,” “aga,” “gaa,” and “ga”) contain “ag” or “ga.” Compared to CF, the difference of importance score in RF among the top 15 features is obviously smaller. This indicates that CF classifier can better reveal the distinct importance of different features in the process of classification, which might be a reason why CF exceeds RF in the classification of cassette exons and constitutive exons.

### 3.4. Comparison with Existing Methods

**Figure 4:**
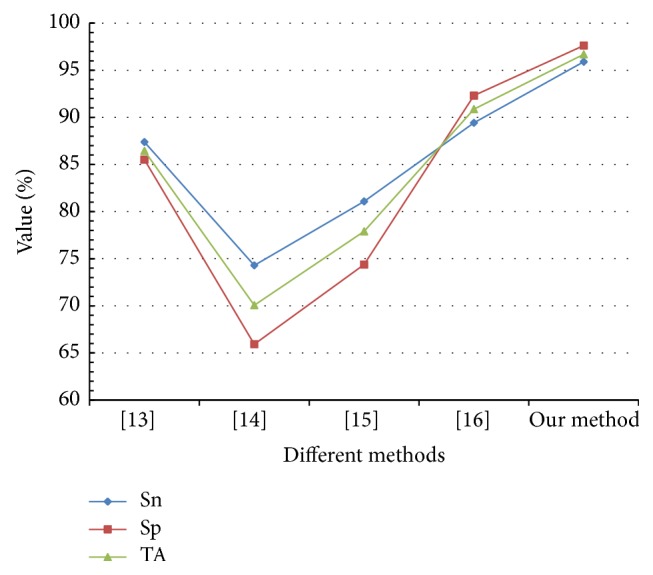
Performance comparison between existing methods and our method.

To demonstrate the superiority of our work, we compare the performance (sensitivity, specificity, and total accuracy) of our method to the methods proposed in [13–16].  Figure 4 shows the classification accuracy of different methods measured by Sn, Sp, and TA. Our method achieved 95.90 percent sensitivity and 97.62 percent specificity, a higher level of accuracy than the other methods.

## 4. Discussion

In this paper, we used a comparative sequence feature analysis that includes length, nucleotide composition (mononucleotide, dinucleotide, and trinucleotide), GC content, termination codon frequency, and splice site strength to distinguish between cassette exons and constitutive exons. The results clearly indicate that cassette exons, the most common AS form of human, have shorter introns, a lower GC content, more termination codons, and weaker splice sites than constitutive exons.

These sequence features are combined in tandem to serve as an input of classifier. We attempted five different classifiers, that is, KNN, SVM, random forest, CForest, and XGBoost, to complement the discrimination task. We use grid search and 10-fold cross-validation to find the optimal parameters for every classification model, trying to make each of them achieve its best performance. Finally, the CForest classifier outperforms the other four models and reaches a total accuracy of 96.69%. With the unbiased variable importance measure supplied in the CForest model, we ranked the importance of all features and displayed the top 15 of them, which is supposed to explore the regulator and contributor of cassette exon events in the human genome. In addition, we presented a comparison of the classification accuracy between our method and other existing methods to validate the superiority of our method in identifying cassette exons. It is obvious that our work provides an improved method for human cassette exons identification.

## Figures and Tables

**Table 1:** Results of sequence feature analysis.

Feature	Mean value of cassette exons	Mean value of constitutive exons	*p* value
Length	142.85	176.39	<2.2*e* − 16
GC content	0.4975	0.5062	0.009886
Termination codon	0.0128	0.0117	0.0006005
5′ splice strength	−13.43874	−12.1145	0.0005285
3′ splice strength	−19.91228	−18.29848	0.0002665

**Table 2:** List of extracted features.

Feature subset	Number of features
Length	1
Mononucleotide	4
Dinucleotide	16
Trinucleotide	64
Termination codon	3
GC content	1
Splice site strength	2
*Total*	*91*

**Table 3:** Classification results of SVM classifier with different kernels.

Kernel	Parameters				*Sensitivity* (%)	*Specificity* (%)	TA (%)
	*C*	*g*	*d*	*r*			
Linear	1				70.23	68.57	69.27
RBF	25	0.000012			**73.23**	**70.34**	**71.69**
Poly	14	0.015623	3		72.26	70.12	71.08
Sigmoid	357	0.000564		0.3	73.27	69.78	71.44

**Table 4:** Parameter optimization details in different classifiers.

Classifier	Parameters	Step size in search	Search range	Optimal value
KNN	*K*	1	1 : 20	7
SVM	*C*	1	1 : 500	25
	*g*	0.000001	10^−6^ : 1	0.000012
Random forest	*ntree*	50	50 : 2000	1000
	*mtry*	1	1 : 91	91
CForest	*ntree*	50	50 : 2000	1050
	*mtry*	1	1 : 91	10
XGBoost	*eta*	0.1	0.1 : 1	0.5
	*max_depth*	1	1 : 10	4

**Table 5:** Classification performance of different classifiers.

Classifier	*Sensitivity* (%)	*Specificity* (%)	TA (%)	AUC
KNN	67.03	75.57	70.45	0.7818
SVM	73.23	70.34	71.69	0.7865
Random forest	71.49	78.72	74.59	0.8411
CForest	95.90	97.62	96.69	0.9954
XGBoost	83.55	85.37	84.44	0.9270

## Additional Points

*Data Availability Statement*. Data used in this paper can be downloaded from http://hexevent.mmg.uci.edu/cgi-bin/HEXEvent/HEXEventWEB.cgi and http://hgdownload.soe.ucsc.edu/downloads.html, and the source code is available at https://sourceforge.net/projects/cassette-exon-prediction/files.
